# Developing a Dementia Care Training Curriculum for Registered Dietitians

**DOI:** 10.1093/geroni/igaa057.1434

**Published:** 2020-12-16

**Authors:** Joy Douglas, Christine Ferguson, Beth Nolan

**Affiliations:** 1 The University of Alabama, Tuscaloosa, Alabama, United States; 2 Positive Approach to Care, Ada, Michigan, United States

## Abstract

Research supports the need for healthcare providers who are trained in providing care to older adults with dementia. However, few training options exist for Registered Dietitians (RDs) seeking dementia care training that is specific to nutrition. The purpose of this project was to adapt an existing dementia care training curriculum to meet the learning needs of RDs. The development team included two experts in dementia training and two RDs with expertise in gerontological nutrition. The new training module was based on the existing Positive Approach to Care™ (PAC) curriculum, which incorporates Kolb’s Experiential Learning Theory and the Adult Experiential Learning Cycle. The development team first identified learning objectives for content that would be relevant to RDs who work with persons living with dementia, and modified components of the existing PAC curriculum to meet these objectives. After a preliminary pilot, the 2-hour program was presented to 20 RDs using a combination of lecture presentation, experiential learning, and skill-building techniques. Participants were provided written materials to reinforce the concepts presented. Participants answered five dementia-specific questions before and after the training, and overall, the average percentage of correct answers improved following the training. Two weeks following the training, participants completed an open-ended survey to provide feedback on the training. Participants responded favorably to the mixed learning formats in the training. When asked to rank their preferred learning methods, participants indicated lecture-based learning and experiential learning as their top preferred methods. These findings indicate that the adapted curriculum may improve dementia knowledge among RDs.

